# PDE7B is involved in nandrolone decanoate hydrolysis in liver cytosol and its transcription is up-regulated by androgens in HepG2

**DOI:** 10.3389/fphar.2014.00132

**Published:** 2014-05-30

**Authors:** Emmanuel Strahm, Anders Rane, Lena Ekström

**Affiliations:** Division of Clinical Pharmaclogy, Department of Laboratory Medicine, Karolinska Institutet, Karolinska University HospitalStockholm, Sweden

**Keywords:** phosphodiesterase 7B, nandrolone decanoate, testosterone, androgens, doping

## Abstract

Most androgenic drugs are available as esters for a prolonged depot action. However, the enzymes involved in the hydrolysis of the esters have not been identified. There is one study indicating that PDE7B may be involved in the activation of testosterone enanthate. The aims are to identify the cellular compartments where the hydrolysis of testosterone enanthate and nandrolone decanoate occurs, and to investigate the involvement of PDE7B in the activation. We also determined if testosterone and nandrolone affect the expression of the PDE7B gene. The hydrolysis studies were performed in isolated human liver cytosolic and microsomal preparations with and without specific PDE7B inhibitor. The gene expression was studied in human hepatoma cells (HepG2) exposed to testosterone and nandrolone. We show that PDE7B serves as a catalyst of the hydrolysis of testosterone enanthate and nandrolone decanoate in liver cytosol. The gene expression of PDE7B was significantly induced 3- and 5- fold after 2 h exposure to 1 μM testosterone enanthate and nandrolone decanoate, respectively. These results show that PDE7B is involved in the activation of esterified nandrolone and testosterone and that the gene expression of PDE7B is induced by supra-physiological concentrations of androgenic drugs.

## Introduction

Testosterone has been therapeutically used for several decades, primarily for androgen replacement therapy in hypogonadal men. Moreover these agents are commonly abused by athletes and sportsmen to improve muscle mass. The abuse of these compounds for cosmetic purposes among non-competing recreational body-builders and non-athletes is a major social concern and has become a growing health problem (Pope and Katz, 1994; Eklof et al., 2003; Sjoqvist et al., 2008; Kanayama et al., 2009). Synthetic analogs such as nandrolone may also have other therapeutic indications such as osteoporosis and aplastic anemia (Frisoli et al., 2005).

Most of the androgenic drugs are available as esters, e.g., testosterone enanthate, nandrolone decanoate, since the androgen itself undergoes first pass metabolism. These drugs have a prolonged depot action due to slow release of the lipophilic steroid ester from the injection site. It is not known which enzymes catalyze the hydrolysis of the esters in order to activate these pro-drugs. We have previously demonstrated that phosphodiesterase 7B (PDE7B) is involved in the hydrolysis and activation of testosterone enanthate (Ekstrom et al., 2011).

PDE7B enzyme belongs to a super-family of 11 members (PDE1-11), all involved in the hydrolyses of intracellular cyclic adenosine monophosphate (cAMP) and cyclic guanosine monophosphate (cGMP) in a variety of cells (Gardner et al., 2000; Hetman et al., 2000; Sasaki et al., 2000). The mechanisms that regulate the expression of PDE genes are not well known. There are two studies showing that glucucorticoids inhibit the PDE mRNA expression (Hermsdorf et al., 1999; Ahlstrom et al., 2005). However, whether anabolic androgenic steroids affect the expression of PDE has not been investigated.

Here we have analyzed if PDE7B is involved in the hydrolysis of nandrolone decanoate using human liver homogenates, microsomes and cytosols. Moreover we have evaluated if PDE7B gene expression is modified by therapeutic and supra-physiological doses of testosterone enanthate and nandrolone decanoate in human liver cells (HepG2) using real-time PCR.

## Materials and methods

### *In-vitro* studies in human liver samples

Five human liver homogenates were obtained from our liver bank (approved by the Ethics Review board in Stockholm). The liver samples were homogenized in 50 mM potassium phosphate from Merck (Darmstadt, Germany) buffer (pH 7.4) and stored at −80°C until use. The incubation was performed at 37°C using different concentrations of nandrolone decanoate from NMI (Lindfield, Australia), 20 μL liver homogenates and Tris-HCl from Sigma-Aldrich Chemie GmbH (Munich, Germany) 50 mM pH 7.4 for a final volume of 250 μL. After a selected incubation time, the reaction mixture was stopped by adding 100 μL acetonitril from Merck and centrifuged 10 min at 3500 rpm prior to injection of 20 μL onto a high performance liquid chromatography system coupled to ultra-violet detection (HPLC-UV). Esterase activity was determined by monitoring the nandrolone formation by analysis on an Agilent 1100 LC system from Agilent Technologies (Palo Alto, CA, USA) system coupled to a UV detector Agilent 1200 sets at a wavelength of 242 nm. The chromatographic separation was performed on a C18 Luna (100 × 4.6, 3 μm) column from Phenomenex Inc. (Torrance, CA, USA) with an isocratic flow of acetonitril/H_2_O (40:60, v:v) at 1.0 mL/min.

In order to determine the cellular compartment where the hydrolysis takes place, cytosols and microsomes were prepared and incubation assays were carried out using the same procedure as for homogenates. Liver pieces were homogenized in buffer (10 mM Na/K phosphate, pH 7.4, containing 1.14% KCl) and then centrifuged (10,000 × g at 4° C for 20 min). The resulting supernatant was further exposed to defined speed centrifugation, whereby a microsomal pellet and a cytosolic fraction were obtained. The pellet was homogenized and mixed with buffer (50 mM potassium phosphate buffer, pH 7.4) and the cytosolic fraction was mixed with dithiothreitol, EDTA, sucrose and glycerol, before storage at −80°C. The protein concentration in liver homogenates, microsomes, and cytosols were performed according to Lowry et al. (1951).

In order to evaluate if PDE7B is involved in the hydrolysis of nandrolone decanoate, inhibition studies were carried out by adding BRL50481 dissolved in DMSO, both from Sigma-Aldrich Chemie GmbH, to the incubation assay (2.5 μL of a 50 mM stock solution for a final concentration of 0.5 mM). The hydrolysis of nandrolone decanoate was also studied in the presence of caffeine from Sigma-Aldrich Chemie GmbH dissolved in the incubation buffer (2.5 μL of a 50 mM stock solution for a final concentration of 0.5 mM).

### Cell culture

All culture media and their ingredients were obtained from Gibco (Life Technologies Ltd, Paisley, UK). Human liver cancer HepG2 cells were cultured in MEM (supplemented with 5% FCS, 1% penicillin/streptomycin, 1% L-glutamine) and maintained in humidified atmosphere at 37°C and 5% CO_2_. Prior to androgen treatment, the HepG2 cells were split and plated in 12-well plates and pre-incubated for 2–3 days. Testosterone enanthate from Sigma-Aldrich Chemie GmbH and nandrolone decanoate were diluted in ethanol 99.5% from Kemetyl (Haninge, Sweden) (stock solution of 1 mM) and added to the cells for 2–48 h at various concentrations (0.01–10 μM). Other compounds were diluted in ethanol for incubation with cells at a final concentration of 1 μM during 2 h; free testosterone and free nandrolone from NMI, estradiol and estradiol cypionate from Sigma-Aldrich Chemie GmbH and R1881 was kindly provided by Professor Anders Bjartell, Karolinska Institute. The non-treated controls were incubated with vehicle only. The experiments were performed in at least four independent experiments. For RNA experiments the cells were harvested with Trizol from Invitrogen (Paisley, UK) and kept at −80°C.

### RNA extraction and cDNA

Total RNA extraction from HepG2 cells was performed using 0.5 mL Trizol per well according to manufacturer's instructions. RNA (0.5 μg) was reverse transcribed into cDNA with hexamer primer using first-strand cDNA synthesis kit from Invitrogen according to the manufacturer's protocol and diluted 20 times in water.

### Real time PCR

The mRNA level of PDE7B in testosterone treated HepG2s was determined by real-time PCR. Beta-actin by Applied Biosystems (Carlsbad, CA, USA) was chosen as an endogenous housekeeping control gene. Quantitative real-time PCR was performed using the 7500 Fast rtPCR from Applied Biosystems. Reaction mixtures contained SYBR green reaction mix from Kapa Biosystems (Woburn, MA, USA), PDE7B primers (as described in Pekkinen et al., 2008), 4 μl cDNA template in a total volume of 15 μl. Thermal cycling conditions included activation at 95°C (10 min) followed by 40 cycles each of denaturation at 95°C (15 s) and annealing/elongation at 60°C (1 min). Each reaction was performed in triplicate and no-template controls were included in each experiment. The untreated sample was employed as a calibrator and the delta CT-formula was used as described in the literature (Livak and Schmittgen, 2001). The gene expression was quantified as the yield of the target gene relative to that of Beta-actin gene.

### Data analysis

The hydrolysis activity, the inhibitory effect in liver sub-cellular fractions as well as the comparison of PDE7B mRNA expression levels between androgen-exposed and non-exposed cells were all compared nonparametric Mann Whitney test. The results are presented as mean ± SD if not stated otherwise. The statistical analyses were performed using GraphPrism Software version 4.03 from GraphPad (San Diego, CA, USA) and values of *P* < 0.05 were considered statistically significant.

## Results

### Activity studies

**Figure 1:**
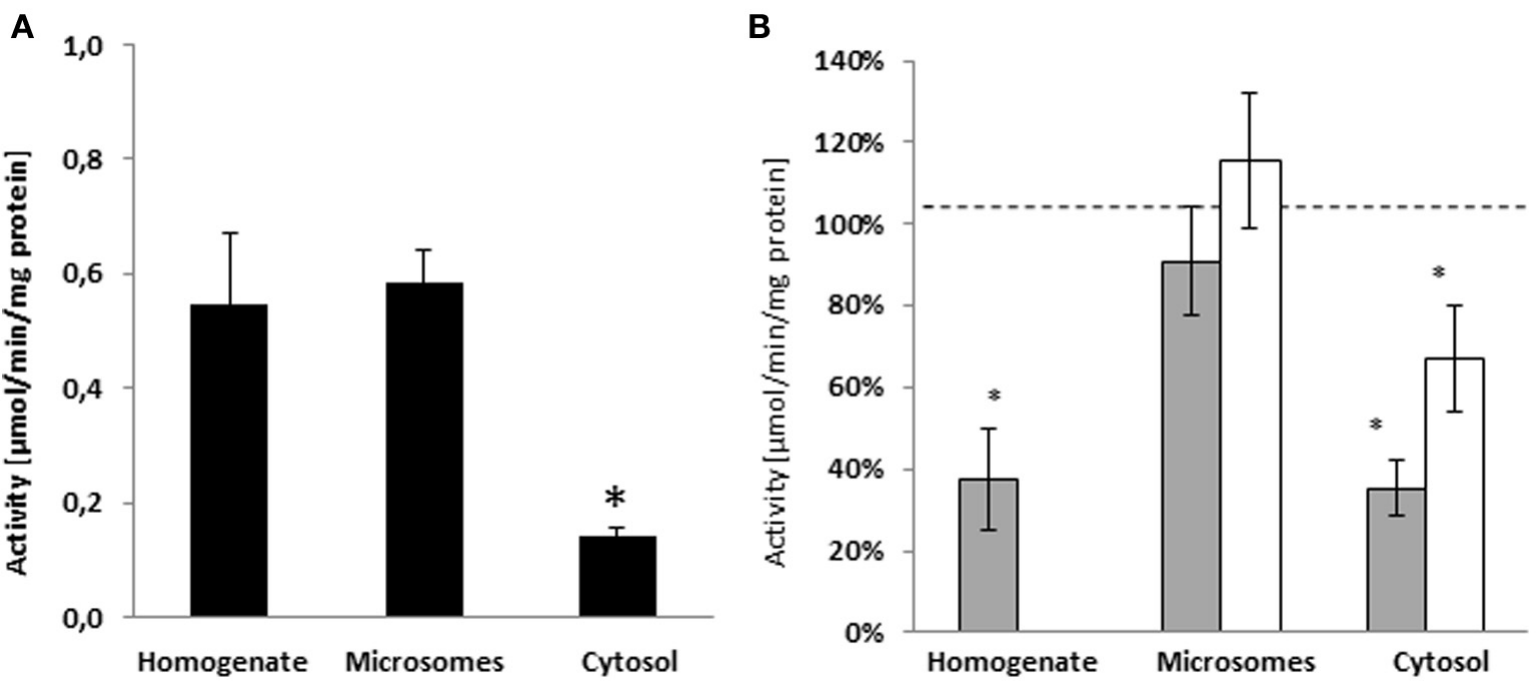
**(A)** Activity of sub-cellular fractions of human liver from Caucasian donors (*n* = 5) on the hydrolysis of nandrolone decanoate. The hydrolysis of nandrolone decanoate was lower in the cytosols as compared to homogenate and microsome fractions. **(B)** The inhibition in hydrolyse activity by PDE7B specific inhibitor (BRL50481) gray bars and a non-selective PDE inhibitor (caffeine) white bars. ^*^p < 0.05.

To assess the cellular compartment where the nandrolone decanoate hydrolysis takes place, we measured the esterase activity in human liver homogenates, microsomes, and cytosols. The hydrolysis of nandrolone decanoate was similar in homogenates (0.55 ± 0.12 μmol/min/mg protein) and microsome (0.58 ± 0.06 μmol/min/mg protein), whereas the activity was significantly lower in cytosols (*p* < 0.045) (0.14 ± 0.02 μmol/min/mg protein) (Figure 1A).

### Inhibitor studies

In a previous study we showed that the hydrolysis of testosterone enanthate in human liver homogenates was inhibited by specific PDE7 inhibitor BRL50481. In order to evaluate if PDE7B is also involved in the hydrolysis of nandrolone decanoate, the specific PDE7B/PDE7A inhibitor BRL50481 was added to the incubation assay. When BRL50481 was added to the incubation assays, the activities were significantly inhibited by 62% in the homogenates (*p* = 0.034) and 65% in the cytosols (*p* = 0.006). In the microsomes, there was no inhibition in esterase activity (Figure 1B).

To further assess if other PDEs may be involved in the hydrolysis of nandrolone decanoate the non-specific PDE7B inhibitor caffeine was tested. The general PDE inhibitor caffeine inhibited the cytosolic hydrolysis (*p* = 0.042) (Figure 1B). There was no significant difference between caffeine and BRL50481 inhibition indicating that caffeine inhibits hydrolysis to the same extent as BRL50481. Caffeine did not inhibit the nandrolone decanoate hydrolysis in microsomes. These results indicate that no other PDEs are involved in the cleavage of steroid esters.

### PDE7B mRNA expression in HepG2 cells

**Figure 2:**
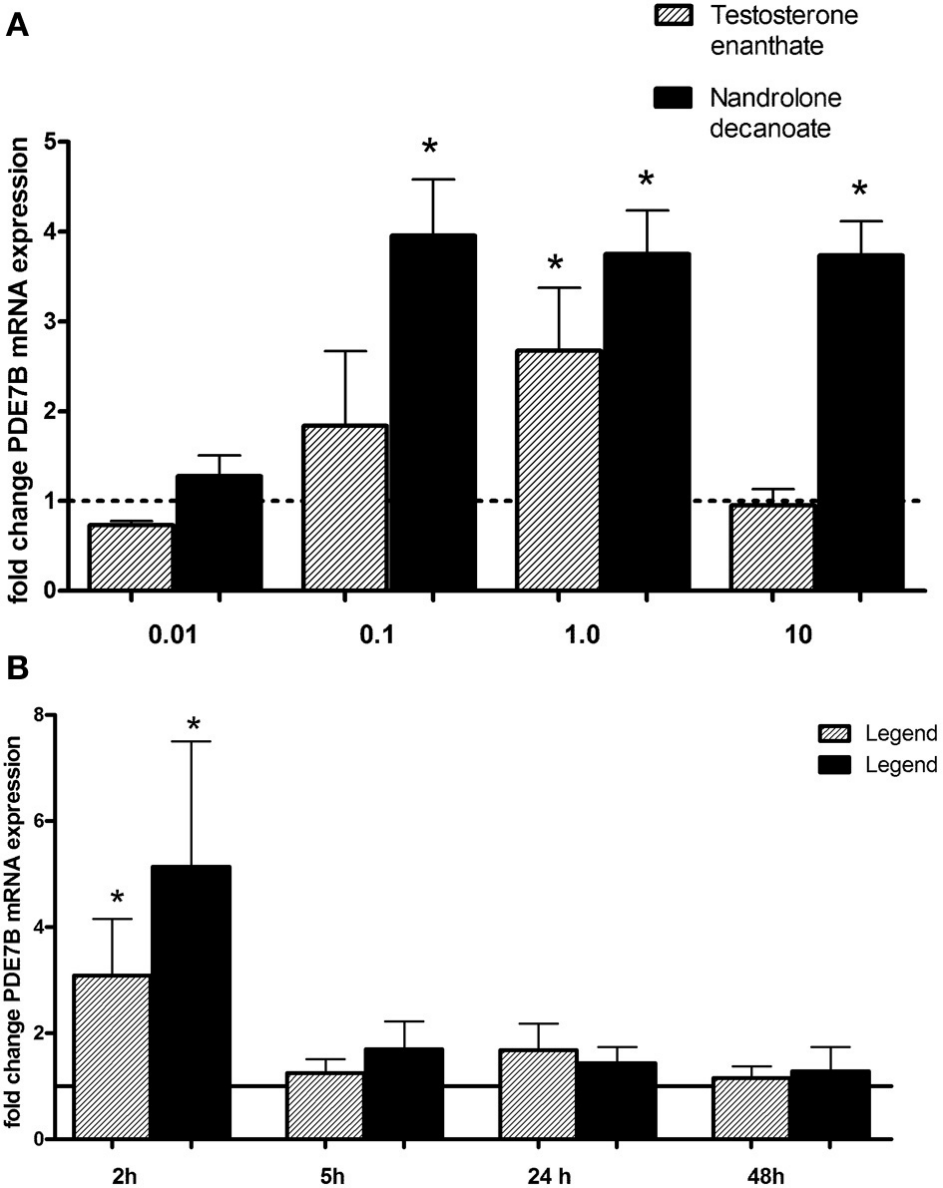
**Relative PDE7B mRNA levels in HepG2 cells after exposure to androgen esters at (A) different concentrations (μM) and incubation time of 2 h and (B) different incubation time (hours) and a concen- tration of 1 μM.** The PDE7B gene expression was induced after 2 h exposure. ^*^p < 0.05.

In order to study if androgen drugs affect the transcriptional activity of PDE7B, testosterone enanthate and nandrolone decanoate, were added at different concentrations (0.01–10 μM) (Figure 2A) for different times point (2–24 h) (Figure 2B) to HepG2 cells. There was no induction at the lowest concentration (0.01 μM) used. Nandrolone decanoate significantly increased PDE7B expression approximately 4-fold at 0.1, 1.0, and 10 μM. Testosterone enanthate significantly increased PDE7B mRNA expression at 1 μM. The gene expression was induced only after 2 h (*p* = 0.049 for testosterone enanthate and *p* = 0.026 for nandrolone decanoate), whereas at 5, 8, and 24 h no induction was observed.

**Figure 3:**
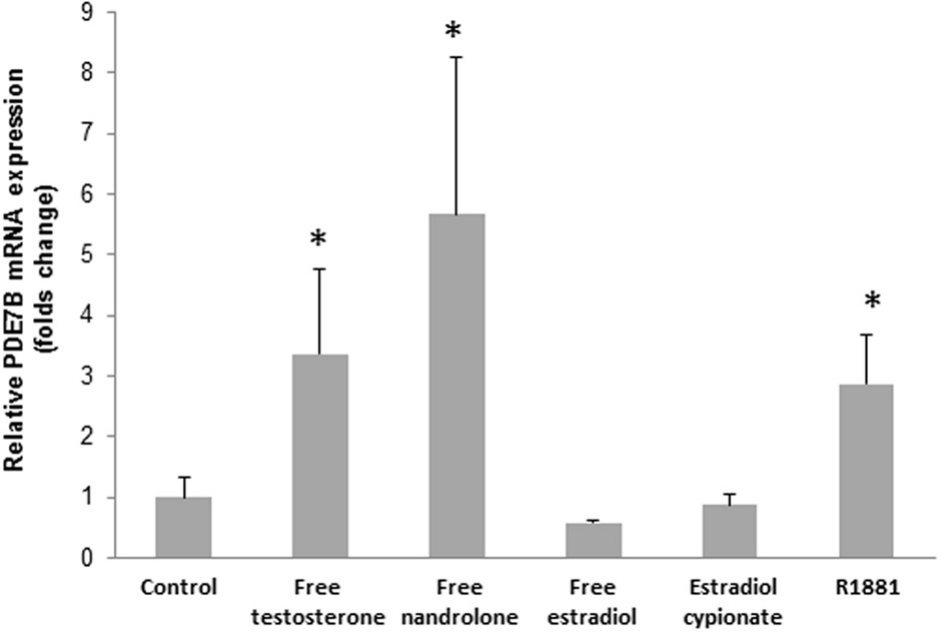
**PDE7B mRNA expression in HepG2 cells after 2h incubation with 1 μM of various hormones (free testosterone, nandrolone, and estrogen, estradiol cypionate, and R1881).** The androgens but not the estrogens induced the PDE7B gene expression. ^*^p < 0.05.

In order to see if testosterone and nandrolone themselves would induce the transcription of PDE7B, free testosterone and nandrolone were added to the cells. The free steroids (1 μM) induced the PDE7B expression after 2 h (*p* = 0.29) (Figure 3) to the same extent as the esterified androgens.

In order to verify that the induction was due to an androgenic effect, rather than an estrogenic effect, we added the synthetic androgen receptor (AR) agonist R1881 and a significant increase in PDE7B expression was observed (*p* = 0.029). Moreover, when estradiol or estradiol cypionate was added to the cells no induction in PDE7B expression was observed (Figure 3).

## Discussion

In a previous *in vitro* study we showed that PDE7B is involved in the hydrolysis of testosterone enanthate. Here we show that PDE7B also activates nandrolone decanoate. Our inhibition studies indicate that the hydrolyze rate of nandrolone decanoate is higher in microsomes than in cytosols. When the specific PDE7 inhibitor BRL50481 was added, the activity was significantly decreased by 65% in the cytosols, whereas in the microsomes no inhibition of the hydrolytic activity was observed. Our results indicate that PDE7B is active in the cytosol compartment. This is in agreement with a previous study showing that the PDE7B specific cAMP hydrolytic activity was found exclusively in the cytosolic extracts of COS-cells (Sasaki et al., 2000). In addition to inhibition of the PDE7B enzyme, BRL50481 also inhibits PDE7A, an enzyme showing 70% sequence homology with PDE7B. In contrast to PDE7B, PDE7A is active both in the cytosol and microsomes (Han et al., 1997; Sasaki et al., 2000). Since we did not find any inhibition in the microsomes, our results further supports that PDE7B and not PDE7A is involved in the hydrolysis of nandrolone decanoate. Our results indicate that approximately 65% of a quarter of the androgen ester hydrolysis is due to PDE7B. The remaining hydrolysis in the cytosol, as well as the high activity found in the microsomes may be catalyzed by other esterases and/or by non-enzymatic activity. Even though only a minor part of the androgen hydrolysis may be catalyzed by PDE7B *in vitro*, PDE7B activity may be of clinical interest since a genetic variation in PDE7B has been shown to be associatied with bioavailibilty of testosterone *in vivo* (Ekstrom et al., 2011).

PDE7B belongs to a superfamily of enzymes, including 11 members (PDE1−11) which often show specific cellular and subcellular distribution. The PDEs are all, to different degree, inhibited by the drugs caffeine and theophylline. When we used caffeine as an inhibitor in our *in vitro* studies, no further inhibition was observed, even though we used high concentrations of caffeine (500 μM) which has been shown in previous studies to inhibit PDEs. These results indicate that no other PDEs are involved in the hydrolysis of nandrolone decanoate.

Here we show for the first time that androgens induce the mRNA expression of PDE7B in HepG2 cells. The induction was observed using androgen concentrations that are in the range of those observed in healthy volunteers after the administration of testosterone enanthate (500 mg) and nandrolone decanoate (125 mg) (Bagchus et al., 2005; Ekstrom et al., 2011). Our results indicate that the transcriptional activation of PDE7B may be of importance after administration of supra-physiological doses of androgens. The reason why PDE7B was induced already after 2 h, but not after 5–48 h is not known, but may be due to a negative feedback on the PDE7B expression. The increase in PDE7B may be accompanied with a decrease in intra cellular level of cAMP which may abolish the androgenic effect since cAMP is known to up-regulate the transcription of PDEs (Manning et al., 1996; Sasaki et al., 2004). Moreover, testosterone and nandrolone are both known to be inactivated by UDP-glucuronosyltransferases 2B enzymes (Kuuranne et al., 2003; Jakobsson et al., 2006) which are highly abundant in HepG2 cells (Nakamura et al., 2008). So it is possible that the bioactive concentration of the androgens rapidly decreases after entering the cells and therefore the effect is diminished.

Testosterone are aromatized to estrogenic metabolites by CYP19 resulting in estrogenic effects in testosterone abusers e.g., gynaecomastia. For nandrolone, the estrogenic effects are mitigated as a result of the drug being a progestin, but nevertheless, effects such as gynaecomastia may still occur at larger doses. In order to confirm that the up-regulation of PDE7B is due to an androgenic effect, rather than an estrogenic effect, we used R1881, a synthetic androgen receptor agonist, free estradiol and estradiol cypionate in our cell experiments. Since R1881, but neither free estradiol nor estradiol cypionate, was shown to increase the transcription of PDE7B we conclude that the induction in PDE7B gene expression is indeed an androgenic effect. However, the core promoter sequence of PDE7B (Accession NM_018945) contains no androgen response element (ARE). It is possible that an ARE is situated further upstreams or that the activated AR may bind and activate other *cis*-acting elements in the PDE7B gene. Moreover, the activated AR may interact with other transcription factors without direct binding to DNA.

PDE7B has been discussed to play a role in various diseases including asthma, chronic lymphocytic leukemia, Alzheimer, schizophrenia (Perez-Torres et al., 2003; Zhang et al., 2008; Ingason et al., 2010; Peiró et al., 2011). PDE7B has also been discussed as an interesting pharmacological target. For example, PDE7B has been proposed as a candidate gene for treatment response to risperidone (Ikeda et al., 2010). Our result indicates that PDE7B may have additional pharmacological roles, i.e., in the metabolism and activation of esterified pro-drugs.

In conclusion here we provide data indicating that PDE7B plays a role in the activation of esterified androgen drugs. Moreover, the expression of PDE7B is induced by supra-physiological concentrations of androgen drugs.

### Conflict of interest statement

The authors declare that the research was conducted in the absence of any commercial or financial relationships that could be construed as a potential conflict of interest.

## References

[B1] Ahlstrom M. Pekkinen M. Huttunen M. Lamberg-Allardt C. (2005). Dexamethasone down-regulates cAMP-phosphodiesterase in human osteosarcoma cells. Biochem. Pharmacol. 69, 267–275 10.1016/j.bcp.2004.09.012 15627479

[B2] Bagchus W. M. Smeets J. M. Verheul H. A. De Jager-Van Der Veen S. M. Port A. (2005). Pharmacokinetic evaluation of three different intramuscular doses of nandrolone decanoate: analysis of serum and urine samples in healthy men. J. Clin. Endocrinol. Metab. 90, 2624–2630 10.1210/jc.2004-1526 15713722

[B3] Eklof A. C. Thurelius A. M. Garle M. Rane A. Sjoqvist F. (2003). The anti-doping hot-line, a means to capture the abuse of doping agents in the Swedish society and a new service function in clinical pharmacology. Eur. J. Clin. Pharmacol. 59, 571–577 10.1007/s00228-003-0633-z 13680032

[B4] Ekstrom L. Schulze J. J. Guillemette C. Belanger A. Rane A. (2011). Bioavailability of testosterone enanthate dependent on genetic variation in the phosphodiesterase 7B but not on the uridine 5'-diphospho-glucuronosyltransferase (UGT2B17) gene. Pharmacogenet. Genomics. 21, 325–332 10.1097/FPC.0b013e328344c5c6 21383644

[B5] Frisoli A. Jr. Chaves P. H. Pinheiro M. M. Szejnfeld V. L. (2005). The effect of nandrolone decanoate on bone mineral density, muscle mass, and hemoglobin levels in elderly women with osteoporosis: a double-blind, randomized, placebo-controlled clinical trial. J. Gerontol. A Biol. Sci. Med. Sci. 60, 648–653 10.1093/gerona/60.5.648 15972619

[B6] Gardner C. Robas N. Cawkill D. Fidock M. (2000). Cloning and characterization of the human and mouse PDE7B, a novel cAMP-specific cyclic nucleotide phosphodiesterase. Biochem. Biophys. Res. Commun. 272, 186–192 10.1006/bbrc.2000.2743 10872825

[B7] Han P. Zhu X. Michaeli T. (1997). Alternative splicing of the high affinity cAMP-specific phosphodiesterase (PDE7A) mRNA in human skeletal muscle and heart. J. Biol. Chem. 272, 16152–16157 10.1074/jbc.272.26.16152 9195912

[B8] Hermsdorf T. Richter W. Dettmer D. (1999). Effects of dexamethosone and glucagon after long-term exposure on cyclic AMP phosphodiesterase 4 in cultured rat hepatocytes. Cell. Signal. 11, 685–690 10.1016/S0898-6568(99)00039-X 10530877

[B9] Hetman J. M. Soderling S. H. Glavas N. A. Beavo J. A. (2000). Cloning and characterization of PDE7B, a cAMP-specific phosphodiesterase. Proc. Natl. Acad. Sci. U.S.A. 97, 472–476 10.1073/pnas.97.1.472 10618442 PMC26687

[B10] Ikeda M. Tomita Y. Mouri A. Koga M. Okochi T. Yoshimur R. (2010). Identification of novel candidate genes for treatment response to risperidone and susceptibility for schizophrenia: integrated analysis among pharmacogenomics, mouse expression, and genetic case-control association approaches. Biol. Psychiatry. 67, 263–269 10.1016/j.biopsych.2009.08.030 19850283

[B11] Ingason A. Giegling I. Cichon S. Hansen T. Rasmussen H. B. Nielsen J. (2010). A large replication study and meta-analysis in European samples provides further support for association of AHI1 markers with schizophrenia. Hum. Mol. Genet. 19, 1379–1386 10.1093/hmg/ddq009 20071346 PMC2838541

[B12] Jakobsson J. Ekström L. Inotsume N. Garle M. Lorentzon M. Ohlsson C. (2006). Large differences in testosterone excretion in Korean and Swedish men are strongly associated with a UDP-glucuronosyl transferase 2B17 polymorphism. J. Clin. Endocrinol. Metab. 91, 687–693 10.1210/jc.2005-1643 16332934

[B13] Kanayama G. Brower K. J. Wood R. I. Hudson J. I. Pope H. G. Jr. (2009). Anabolic-androgenic steroid dependence: an emerging disorder. Addiction. 104, 1966–1978 10.1111/j.1360-0443.2009.02734.x 19922565 PMC2780436

[B14] Kuuranne T. Kurkela M. Thevis M. Schanzer W. Finel M. Kostiainen R. (2003). Glucuronidation of anabolic androgenic steroids by recombinant human UDP-glucuronosyltransferases. Drug Metab. Dispos. 31, 1117–1124 10.1124/dmd.31.9.1117 12920167

[B15] Livak K. J. Schmittgen T. D. (2001). Analysis of relative gene expression data using real-time quantitative PCR and the 2(-Delta Delta C(T)) Method. Methods 25, 402–408 10.1006/meth.2001.1262 11846609

[B16] Lowry O. H. Rosebrough N. J. Farr A. L. Randall R. J. (1951). Protein measurement with the Folin phenol reagent. J. Biol. Chem. 193, 265–275 14907713

[B17] Manning C. D. McLaughlin M. M. Livi G. P. Cieslinski L. B. Torphy T. J. Barnetti M. S. (1996). Prolonged beta adrenoceptor stimulation up-regulates cAMP phosphodiesterase activity in human monocytes by increasing mRNA and protein for phosphodiesterases 4A and 4B. J. Pharmacol. Exp. Ther. 276, 810–818 8632354

[B18] Nakamura A. Nakajima M. Yamanaka H. Fujiwara R. Yokoi T. (2008). Expression of UGT1A and UGT2B mRNA in human normal tissues and various cell lines. Drug Metab. Dispos. 36, 1461–1464 10.1124/dmd.108.021428 18480185

[B19] Peiró A. M. Tang C. M. Murray F. Zhang L. Brown L. M. Chou D. (2011). Genetic variation in phosphodiesterase (PDE) 7B in chronic lymphocytic leukemia: overview of genetic variants of cyclic nucleotide PDEs in human disease. J. Hum. Genet. 56, 676–681 10.1038/jhg.2011.80 21796143 PMC3833258

[B20] Pekkinen M. Ahlstrom M. E. Riehle U. Huttunen M. M. Lamberg-Allardt C. J. (2008). Effects of phosphodiesterase 7 inhibition by RNA interference on the gene expression and differentiation of human mesenchymal stem cell-derived osteoblasts. Bone 43, 84–91 10.1016/j.bone.2008.02.021 18420479

[B21] Perez-Torres S. Cortes R. Tolnay M. Probst A. Palacios J. M. Mengod G. (2003). Alterations on phosphodiesterase type 7 and 8 isozyme mRNA expression in Alzheimer's disease brains examined by in situ hybridization. Exp. Neurol. 182, 322–334 10.1016/S0014-4886(03)00042-6 12895443

[B22] Pope H. G. Jr. Katz D. L. (1994). Psychiatric and medical effects of anabolic-androgenic steroid use. A controlled study of 160 athletes. Arch. Gen. Psychiatry 51, 375–382 10.1001/archpsyc.1994.03950050035004 8179461

[B23] Sasaki T. Kotera J. Omori K. (2004). Transcriptional activation of phosphodiesterase 7B1 by dopamine D1 receptor stimulation through the cyclic AMP/cyclic AMP-dependent protein kinase/cyclic AMP-response element binding protein pathway in primary striatal neurons. J. Neurochem. 89, 474–483 10.1111/j.1471-4159.2004.02354.x 15056290

[B24] Sasaki T. Kotera J. Yuasa K. Omori K. (2000). Identification of human PDE7B, a cAMP-specific phosphodiesterase. Biochem. Biophys. Res. Commun. 271, 575–583 10.1006/bbrc.2000.2661 10814504

[B25] Sjoqvist F. Garle M. Rane A. (2008). Use of doping agents, particularly anabolic steroids, in sports and society. Lancet 371, 1872–1882 10.1016/S0140-6736(08)60801-6 18514731

[B26] Zhang L. Murray F. Zahno A. Kanter J. R. Chou D. Suda R. (2008). Cyclic nucleotide phosphodiesterase profiling reveals increased expression of phosphodiesterase 7B in chronic lymphocytic leukemia. Proc. Natl. Acad. Sci. U.S.A. 105, 19532–19537 10.1073/pnas.0806152105 19033455 PMC2614795

